# Two, Six, and Twelve-Month Dropout Rate and Predictor Factors After a Multidisciplinary Residential Program for Obesity Treatment. A Prospective Cohort Study

**DOI:** 10.3389/fnut.2022.851802

**Published:** 2022-05-27

**Authors:** Simone Perna, Majeda Salman, Clara Gasparri, Alessandro Cavioni, Milena Anna Faliva, Francesca Mansueto, Maurizio Naso, Zaira Patelli, Gabriella Peroni, Alice Tartara, Antonella Riva, Giovanna Petrangolini, Mariangela Rondanelli

**Affiliations:** ^1^Department of Biology, College of Science, University of Bahrain, Sakhir, Bahrain; ^2^Department of Mathematics, College of Science, University of Bahrain, Sakhir, Bahrain; ^3^Endocrinology and Nutrition Unit, Azienda di Servizi alla Persona ‘Istituto Santa Margherita', University of Pavia, Pavia, Italy; ^4^Research and Development Unit, Indena, Milan, Italy; ^5^Istituto di Ricovero e Cura a Carattere Scientifico (IRCCS) Mondino Foundation, Pavia, Italy; ^6^Department of Public Health, Experimental and Forensic Medicine, University of Pavia, Pavia, Italy

**Keywords:** obesity, dropout, predictor factors, weight loss, multidisciplinary program

## Abstract

**Introduction:**

The aim of the present study was to assess the dropout rate at 2, 6, and 12 months after an inpatient multidisciplinary residential program (MRP) for the treatment of obesity. Furthermore, this study assessed anthropometric and biochemical predictors associated with the dropout.

**Methods:**

Adult and elderly patients (age 59 ± 14 years) with obesity had undergone an MRP, were followed up from 2 to 12 months. Biochemical and anthropometric markers have been assessed at the beginning of the follow-up period after the MRP.

**Results:**

The study enrolled 178 subjects, 117 women and 61 men. The overall dropout rate at 2 months was 21.3%, after 6 months was 44.4%, and after 1 year was 68.5%. There was no difference by gender recorded. Furthermore, patients under medical treatment with psychiatric disorders did not show an association with the dropout rate. Patients with a higher level of body mass index (BMI) at the discharge of MRP showed +48% of dropout at 6 months. After the MRP, the baseline values of uricemia and white blood cells (WBCs) resulted as predictors of dropout at 2 months (*p* > 0.05). Furthermore, the excess percentage of fat mass lost during the MRP was associated with the risk of dropout at 2, 6, and 12 months (*p* > 0.05).

**Conclusion:**

The MRP for obesity is an opportunity for losing weight for patients with established criteria. The future challenge will be addressing the best strategic plans in order to reduce the dropout rate after this intervention. Investigating deeply the main predictors could be an opportunity to improve the long-term efficacy of MRP.

## Introduction

According to the WHO, obesity is one of the main public health problems in the world (1). In fact, higher body weight is associated with a higher incidence of a number of conditions, including diabetes mellitus, cardiovascular disease, and non-alcoholic fatty liver disease, and with an increased risk of disability and mortality (2–4). Obesity negatively affects heart function, increases risk factors for coronary heart disease, and is an independent risk factor for cardiovascular disease (American Heart Association).

Evidence suggests that, even without reaching an ideal weight, moderate weight loss may be beneficial in terms of reducing certain risk factors, such as high blood pressure (2). Some studies on dietary and behavioral treatments, however, have shown that maintaining a weight loss is difficult (2, 5).

The treatment for obesity must include a low-calorie diet, the introduction of regular physical activity, and measures that promote behavioral change (4, 6). Also, after weight reduction, long-term measures are needed for its maintenance (4).

Identifying patients at risk of non-adherence to long-term follow-up will contribute to both the effectiveness and cost-effectiveness of weight loss interventions (7, 8).

The literature on the dropout rate in the treatment of obesity is heterogeneous, with data ranging from 10 to 80% at 12 months depending on the types of program (7). Intervention studies have reported an average dropout rate of over 40% within the first 12 months (8, 9).

Regarding the major predictor of adherence, the greater weight loss during the first month of treatment and participation in a higher percentage of encounters are strongly associated with greater weight loss at the end of treatment and the 1-year follow-up (10). Furthermore, the weight loss at the beginning of the rehabilitation program is a crucial determinant of abandonment, i.e., a poor initial response is a predictor of abandonment of treatment (11–13). There are currently no studies demonstrating the efficacy of MRP intervention after hospitalization in terms of maintenance of efficacy and dropout risk.

The aim of the present study is to assess the dropout rate at 2, 6, and 12 months after an inpatient multidisciplinary residential program (MRP) for the treatment of obesity. Furthermore, this study assessed anthropometric and biochemical predictors associated with the dropout.

## Methods

### Study Protocol

This is a prospective cohort study in which obese participants, who had undergone MRP in the Metabolic Rehabilitation Unit of the Azienda di Servizi alla Persona, Istituto Santa Margherita, University of Pavia (27100 Pavia, Italy), were evaluated from the time of the discharge of MPD intervention to 1 year-follow up. The baseline was after the MRP discharge and the follow-up was established at 2, 6, and 12 months.

Previously, patients underwent an MRP, which included a hypocaloric diet, physical exercise, and psychological support. Under hospitalization, participants were administered with a restrictive hypo-caloric diet under hospitalization for a maximum period of 3 months. Body weight reduction was induced by a low-energy mixed diet (55% carbohydrates, 30% lipids, and 15% proteins) providing 600 kcal less than individual energy requirements based on the measured TEE. The intervention was designed to achieve a weight loss of 0.5–1 kg per week; this type of diet is considered to be a low-risk intervention (14). To optimize compliance, dietary instructions were reinforced each week by a dietician. A personalized hypocaloric diet to be followed at home was provided to each patient at discharge. Weight loss expectations (0.5 kg per week) were discussed with the multidisciplinary team at discharge.

The exercise program was based on the physical activity recommendations for adults proposed by WHO (15), on progression models in strength and aerobic training for healthy adults. Limited information regarding the ideal exercise model for morbidly obese adults exists, so the intervention was based on combined strength and aerobic training (i.e., a concurrent training protocol), as previous findings in obese adults displayed important benefits when both strength and aerobic exercise are implemented in the same session (16) of 60 min of 5 days a week and more than 10,000 steps per day. Physical activity was individualized and conducted every day by each subject with the help of a qualified and properly trained physiotherapist. Psychological management is based on the enhanced cognitive behavior therapy (CBT-E) approach, which is considered the most valid methodology for the treatment of eating disorders (17). Psychological support during the MRP had the dual purpose of defining the presence of eating disorders and providing psychoeducation and strategies for adhering to the new diet. Individual interviews have been carried out weekly with the aim of reducing psychopathology, if present, investigating the factors of maintenance of the disorder and carrying out a cognitive restructuring. In addition, multidisciplinary group meetings are held with an expert dietician to identify functional strategies for managing the diet once back home.

### Ethics Committee

The study design was approved by the ethics committee of the University of Pavia, and individual written informed consent was obtained from each participant. Data were gathered from 1 March 2016 to 1 March 2021. Outcomes were assessed at 2, 6, and 12 months after discharge during outpatient visits.

### Participants

Sample size calculation has been performed with the following calculator: https://riskcalc.org/samplesize/ in 137 patients with 2-side significance level at 0.05, power (1-beta) 0.8. Eligible participants were aged >18 years with body mass index (BMI) ≥30 Kg/m2 with one or more metabolic comorbidities (type 2 diabetes mellitus, dyslipidemia, high blood pressure, hyperuricemia, etc.). Patients with acute psychiatric comorbidities have been excluded. The check-up outpatient visits were carried out at 2 (T1), 6 (T2), and 12 (T3) months after discharge. During each visit, the patients' adherence to the diet was evaluated through the measure of BMI and body composition parameters [fat mass (FM), fat-free mass (FFM), and visceral adipose tissue (VAT)].

### Anthropometric Measurements

After MRP discharge, the anthropometric parameters, such as body weight, waist, and hip circumference, were measured during each outpatient visit. Bodyweight was measured to the nearest 0.1 kg, using a precision scale; participants wore light clothing, no shoes, and a standardized method was used (18). The waist was measured at the midpoint between the top of the hip bone (iliac crest) and lowest rib, using a standardized method.

### Body Composition Assessment by Double X-Ray Densitometry

After MRP discharge body composition (FFM, FM, visceral fat mass) was determined by dual-energy X-ray absorptiometry (DXA), using a Lunar Prodigy DXA (GE Medical Systems). The *in vivo* CVs were 0.89% for whole body fat (FM) and 0.48% for FFM. The Skeletal Muscle Index (SMI) was taken as the sum of the fat-free soft tissue mass of arms and legs divided by height^2^. (19). VAT volume was estimated using a constant correction factor (0.94 g/cm3) (20). Subcutaneous abdominal fat was defined as the difference between android fat and visceral fat. The *in vivo* CVs were 0.89 and 0.48% for FM and FFM, respectively (21).

### Biochemical Analysis

After MRP discharge the blood samples were collected at the end of the MRP (baseline). In particular, nutritional status, lipid profile, glycemic profile, and status of inflammation were assessed. Serum iron, lipids, uric acid, creatinine, and calcium were measured by enzymatic-colorimetric assay (Abbott Laboratories). PCR, Transferrin, Apo A1, and Apo B were determined by immunoturbidimetry (Roche). ESR was measured by the Westergren method using a Diesse Analyzer, blood electrolytes by indirect ISE potentiometry (Abbott Laboratories), ionized Calcium by selective electrode potentiometry, and insulin by Electro-chemiluminescence immuno-assay (ECLIA) (Roche Diagnostics). Blood glucose, aspartate aminotransferase (AST), and alanine aminotransferase (ALT) were analyzed by Enzymatic UV Assay (Abbott Laboratories) and CBC by differential blood cell counter. Insulin resistance was evaluated using the Homeostasis Model Assessment (HOMA) (22).

### Statistical Analysis

Statistical analyses were carried out using the programs SPSS version 26.0 and JASP version 14.1. Continuous variables were tested for normality using Kolmogorov–Smirnov's test at the 0.05 level of significance. Descriptive statistics for normally distributed variables were reported through the mean and SD (Mean ± SD) and through the median and inter-quartile range (MD ± IQR) otherwise.

Categorical variables were described using frequencies of counts and percentages. The dropout rates at 2, 6, and 12 months were reported with percentages relative to the total sample. In the determination of the predicting factors to the dropout, we first tested the mean difference in anthropometric measures at baseline between the dropout and non-dropout groups at 12 months using the independent Student's *t*-test. Second, we used binary logistic regression to determine the association between the anthropometric and biochemical predictors on the dropout at 2, 6s, and 12 months. Predictors are considered significant, if their *p*-value < 0.05.

We estimated both the unique univariable associations between each individual predictor [i.e., crude hazard ratios (HR)] and the multivariable associations between the predictors and time to dropout. The HR indicates the relative risk of treatment dropout when all remaining factors in the model are adjusted for and is interpreted in a similar manner to the adjusted odds ratio in logistic regression.

## Results

Tables 1, 2 show the baseline characteristics for anthropometric measurements and biochemical markers. The mean age was 59 ± 14 years. The mean of BMI was 41.35 ± 6.31 kg/m^2^: for men 41.42 ± 5.52 kg/m^2^ and for women 41.31 ± 6.72 kg/m^2^.

**Table 1:** Baseline anthropometric measurements.

**Variables**	**Women (*n* = 117)**	**Men (*n* = 61)**	**Total (178)**
	**Mean ±*SD***	**Mean ±*SD***	**Mean ±*SD***
Age (years)	62 ± 13	55 ± 15	59 ± 14
Weight (kg)	99.1 ± 17.8	120.5 ± 18.4	106.5 ± 20.7
Δ weight (kg)	−5.6 ± 2.9	−9.5 ± 4.9	−7 ± 4.1
BMI (kg/m2)	41.31 ± 6.72	41.42 ± 5.52	41.35 ± 6.31
Δ BMI (kg/m2)	−2.33 ± 1.1	−3.28 ± 1.64	−2.66 ± 1.38
Waist (cm)	119.3 ± 12.9	132.2 ± 11.4	123.7 ± 13.8
Δ waist (cm)	−5.6 ± 3.5	−7.8 ± 3.5	−6.4 ± 3.7
FFMI (g)	19005.65 ± 2266.7	20786.24 ± 3356.59	19602.67 ± 2801.64
FMI (g)	18948.06 ± 4493.02	15443.44 ± 3347.83	17766.03 ± 4453.73
FM (%)	49.5 ± 4.8	41.9 ± 4.7	46.9 ± 6
SMI (g)	8.99 ± 1.13	10.03 ± 1	9.33 ± 1.19
VAT (g)	1,703 ± 576	2,842 ± 995	2,061 ± 903

**Table 2:** Baseline biochemical characteristics.

**Variable**	**Women (*n* = 117)**	**Men (*n* = 61)**	**Total**
	**Mean ±*SD***	**Mean ±*SD***	**Mean ±*SD***
Vit D (ng/ml)	32.5 ± 13.6	32.9 ± 16.6	32.6 ± 14.6
ESR (mm/h)	28 ± 18	21 ± 17	25 ± 18
Glycemia (mg/dl)	88 ± 14	90 ± 15	89 ± 14
Chol Tot (mg/dl)	166 ± 33	150 ± 32	161 ± 33
TRG (mg/dl)	114 ± 38	132 ± 51	120 ± 43
Insulin (mcU/ml)	8.77 ± 8.66	13.50 ± 10.15	10.65 ± 10.06
Uricemia (mg/dl)	6.1 ± 1.6	7.1 ± 1.6	6.4 ± 1.6
Creatinine (mg/dl)	0.88 ± 0.3	1.1 ± 0.38	0.95 ± 0.34
Azotemia (mg/dl)	41 ± 15	39 ± 17	40 ± 16
TSH (mcIU/ml)	2.293 ± 2.044	1.738 ± 1.036	2.125 ± 1.813
Homocysteine (μmol/l)	15.92 ± 4.85	17.1 ± 5.12	16.3 ± 4.95
RBC (M/ul)	4.57 ± 0.45	4.8 ± 0.47	4.65 ± 0.47

The study enrolled 178 subjects (65.73%), who were women. As shown in Table 3, there was an early dropout after 2 months of therapy by a rate of almost 21% in both men and women. The dropout rate by women after 6 months was 43.6% and by men was 45.9%.

**Table 3:** Dropout rate at 2, 6, and 12 months.

**Subject**	**Up to 2 months**	**Up to 6 months**	**Up to 12 months**
**Women**			
100% (117)	21.4% (25)	43.6% (51)	70.0% (82)
**Men**			
100% (61)	21.3% (13)	45.9% (28)	65.6% (40)
**Total**			
100% (178)	21.3% (38)	44.4% (79)	68.5% (122)

After 12 months 70% of women and 65.6% of men abandon the treatment. There was no significant association between gender and the period of which the dropout took place (*p* = 0.581, NS).

Table 4 shows that psychiatric comorbidities did not affect the dropout rate over time. Patients with psychiatric comorbidities had the same rate of dropout at 2, 6, and 12 months.

**Table 4:** Dropout rate at 2, 6, and 12 months in patients with psychiatric comorbidities.

**Patients with psychiatric comorbidities**	**Dropout at 2 months**	**Dropout at 6 months**	**Dropout at 12 months**
Yes	28.9%	27.5%	25.2%
No	71.1%	72.5%	74.8%

Table 5 reports the marginal effect of these predictors on 12 months dropout was supported by the almost equal odds ratio provided by the simple logistic regression. Patients with a higher level of BMI at the discharge of MRP showed +48% of dropout risk at 6 months. Table 6 shows that after the MRP intervention, the baseline values of uricemia (*p* > 0.05), and white blood cells (WBCs) (*p* > 0.05) resulted as main predictors of dropout at 2 months. Furthermore, as shown in Table 7, the excess of Δ% FM lost during the MRP was associated with the risk of dropout at 2, 6, and 12 months.

**Table 5:** Binary regression of dropout risk at 2, 6, and 12 months considering the anthropometric values after the discharge of MRP.

	**Dropout at 2 months**		**Dropout at 6 months**		**Dropout at 12 months**	
	**Sig**.	**Exp (B)**	**Sig**.	**Exp (B)**	**Sig**.	**Exp (B)**
Psychiatric Comorbidities	0.093	2.303	0.245	1.632	0.331	1.642
Gender	0.529	1.859	0.208	0.364	0.333	0.437
Age (years)	0.859	1.004	0.337	0.984	0.134	0.970
hospitalization (days)	0.463	0.991	0.378	0.993	0.087	0.988
Weight (kg)	0.798	0.992	0.555	0.985	0.755	0.992
BMI (kg/m2)	0.521	1.150	**0.030	1.482	0.151	1.328
FFMI	0.695	1.000	0.126	1.000	0.095	1.000
FMI	0.884	1.000	0.056	1.010	0.155	1.000
SMI	0.304	0.710	0.674	0.910	0.881	0.962
VAT (g)	0.712	1.000	0.087	1.001	0.835	1.000

** *in bold p-value < 0.05*.

**Table 6:** Binary regression of dropout risk at 2, 6, and 12 months considering the biochemical measures after the discharge of MRP.

	**Dropout at 2 months**		**Dropout at 6 months**		**Dropout at 12 months**	
	**Sig**.	**Exp (B)**	**Sig**.	**Exp (B)**	**Sig**.	**Exp (B)**
Psychiatric comorbidities	0.902	1.183	0.722	1.404	0.399	0.390
Gender	0.061	20.450	0.148	0.181	0.059	0.042
Age (years)	0.077	0.909	0.617	0.981	0.368	0.957
hospitalization (days)	0.280	0.969	0.579	1.013	0.125	0.957
Vit D T1 (ng/ml)	0.098	1.063	0.980	1.001	0.328	0.970
Glycemia T1 (mg/dl)	0.337	1.032	0.839	1.006	0.617	0.982
Uricemia T1 (mg/dl)	*0.024	0.346	0.864	1.055	0.810	1.098
Azotemia T1 (mg/dl)	0.115	1.099	0.887	0.993	0.177	0.912
Creatinine T1 (mg/dl)	0.084	0.002	0.073	3.655	0.072	6.741
ESR T1 (mm/h)	0.659	0.987	0.419	0.981	0.776	1.008
Chol Tot T1 (mg/dl)	0.787	1.006	0.489	1.011	0.056	0.962
TRG T1 (mg/dl)	0.276	0.985	0.915	0.999	0.232	0.982
TSH T1 (mcIU/ml)	0.610	0.844	0.167	0.661	0.875	1.056
Insulin T1 (mcU/ml)	0.206	0.918	0.854	1.011	0.879	1.011
Homocysteine T1 (μmol/l)	0.108	1.286	0.511	1.072	0.593	1.064
RBC T1 (M/ul)	0.103	6.563	0.743	0.736	0.647	0.592
WBC T1 (K/ul)	*0.038	2.136	0.138	1.435	0.086	1.792

* *in bold p-value < 0.05*.

**Table 7:** Binary regression of dropout risk at 2, 6, and 12 months considering the anthropometric markers changed during the MRP.

	**Dropout at 2 months**		**Dropout at 6 months**		**Dropout at 12 months**	
	**Sig**.	**Exp (B)**	**Sig**.	**Exp (B)**	**Sig**.	**Exp (B)**
Psychiatric comorbidity	0.049	3.038	0.144	1.990	0.131	2.468
Gender	0.407	0.566	0.180	0.495	0.005	0.195
Age (years)	0.535	1.015	0.086	0.969	0.030	0.957
recovery period (days)	0.355	0.983	0.243	0.992	0.084	0.988
Δ BMI (kg/m2)	0.087	0.576	0.120	0.665	0.154	0.622
Δ waist (cm)	0.602	1.047	0.890	0.990	0.693	1.030
Δ FM (%)	0.003**	1.914	0.001**	1.900	**0.005**	1.741
Δ VAT (g)	0.274	0.999	0.705	1.000	0.206	0.999
Δ SMI	0.227	1.669	0.941	0.974	0.258	0.637

** *in bold p-value < 0.05. Delta change is given by the effect of MRP*.

**Figure 1:**
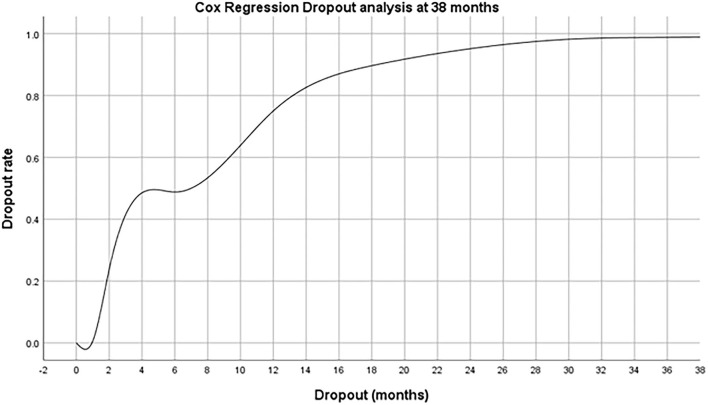
Cox regression dropout survival analysis at 38 months after MRP.

No additional statistically significant predictive factors for dropout have been recorded at 2, 6, and 12 months. Figure 1 shows the cox regression dropout analysis at 38 months after MRP.

## Discussion

The study was performed to understand the “post efficacy” of an MRP for obesity in terms of dropout rate and its predictors. This is the first study in the literature reporting dropout data after an MRP for obesity.

First, this study showed an early dropout rate at 2 months of therapy in 21% of all patients, while 44% after 6 months, and around 69% at 12 months.

Our results showed that the dropout rate in patients following an MRP program shows a similar trend compared with normal outpatient program follow-up without a previous MRP, as reported by Perna et al., that reported a drop-out rate of 68% (23).

Concerning the main anthropometric predictor factors of dropout, the current analysis concluded that the BMI at 6 months of discharge of MRP represents the main predictor. Patients with higher BMI at the discharge of MRP showed a +48% of dropout rate at 6 months. This critical point should be addressed in future studies since the MRP for obesity could be an opportunity to lose weight for a specific cohort of patients with specific characteristics, not for all. This data has also been found in a recent study that explored sociodemographic and clinical characteristics as possible predictors of dropping out of the weight loss program, concluding that initial weight loss at 1 month made the strongest contribution to weight loss prediction percentage after 12 months (24, 25).

Other two recent studies demonstrated that greater distance to the clinic and greater patient's initial BMI were associated with higher attrition, specifically in patients with post bariatric surgery interventions (26, 27).

In a recent study by Dalle Grave et al., it was found that the dropout rate increased by 10% for any point of increase in the expected BMI loss (8). Even another study (25) concluded that dissatisfaction with results is a major cause of dropout (25% of dropouts). From this perspective, it is important to note that satisfaction with the body weight achieved during treatment is significantly associated with better weight maintenance (28).

The present study showed another interesting situation: patients with a higher loss of FM during the MRP have a higher risk of dropout at 2, 6, and 12 months. The excess of FM loss seems to be one important risk factor for dropout overtime. The causes could be unknown, but we should highlight that the excess of FM has been described as associated with fatigue and several hormonal changes that could lead to a chronic depression state (29).

Furthermore, this study showed that uricemia and WBCs have been associated with the dropout at 2 months. Although there is no specific scientific evidence regarding the real role of uricemia and WBC on the dropout rate, it is worth noting that similar data were shown in the study of Perna et al., still, the causes are unknown (23).

Finally, this study studied the effect of psychiatric comorbidities on the dropout rate after the MRP, showing that there is no effect, regarding the dropout at 2, 6, and 12 months.

In general, mood disorders may impact the long-term outcomes of a weight loss program. As in bariatric surgery for weight loss, available evidence suggests that mental disorders are associated with poorer weight loss following the intervention (29).

During the previous MRP, psychological management based on the CBT-E approach was carried out. A recent study confirmed the significant bi-directional associations found between a weight loss intervention and mental health/quality of life, indicating the importance of considering mental health and quality of life as part of any weight loss intervention for older adults (30).

The main limitation of the study was the imbalance between men and women. The motivation of the patients was assessed through psychological support; however, most of the patients did not continue the psychological support after discharge, so this can represent a limitation. On the contrary, since the data showed that participants who lost more weight during the treatment period had a better chance of 12 months adherence to the instructions, there is the possibility that program adherence may be biased by some people who had a higher degree of determination to lose weight even before entering the treatment program. An additional result of our study was that patients that had an increase of ΔFM% during MRP, showed an increase of +14% of dropout overtime. The excessive loss of FM during the MRP could affect the attrition of future weight management treatments as shown by Colombo et al. (12).

## Conclusion

The multidisciplinary residential program for obesity is an opportunity for losing weight for patients with established criteria. The future challenge will be addressing the best strategic plans in order to reduce the dropout rate after this intervention. Investigating deeply the main predictors could be an opportunity to improve the long-term efficacy of this intervention.

## Data Availability Statement

The raw data supporting the conclusions of this article will be made available by the authors, without undue reservation.

## Author Contributions

MR and SP: conceptualization. MS and SP: methods and formal analysis. CG and GaP: data curation. CG, MS, MF, and MN: writing—original draft. AC, FM, ZP, and AT: writing—review and editing. AR and GiP: supervision. All authors have read and approved the manuscript.

## Conflict of Interest

AR and GiP are employed by Indena S.p.a. The remaining authors declare that the research was conducted in the absence of any commercial or financial relationships that could be construed as a potential conflict of interest.

## Publisher's Note

All claims expressed in this article are solely those of the authors and do not necessarily represent those of their affiliated organizations, or those of the publisher, the editors and the reviewers. Any product that may be evaluated in this article, or claim that may be made by its manufacturer, is not guaranteed or endorsed by the publisher.
